# 3D-printed polycaprolactone combined with cartilage acellular matrix tissue engineered cartilage scaffold manufactured by low-temperature deposition manufacturing

**DOI:** 10.3389/fbioe.2025.1604515

**Published:** 2025-07-23

**Authors:** Zhen Song, Xulong Zhang, Yihao Xu, Jingyuan Ji, Wei Sun, Fei Fan, Jianjun You, Yuan Pang

**Affiliations:** ^1^Department of Plastic Surgery, Henan Provincial People’s Hospital, Zhengzhou, China; ^2^Department of Rhinoplasty, Plastic Surgery Hospital, Chinese Academy of Medical Sciences and Peking Union Medical College, Beijing, China; ^3^Department of Mechanical Engineering, Biomanufacturing Center, Tsinghua University, Beijing, China

**Keywords:** tissue engineered cartilage, cartilage acellular matrix, polycaprolactone, low-temperature deposition manufacturing, composite scaffold

## Abstract

**Introduction:**

Tissue-engineered cartilage provides an alternative for tissue repair and reconstruction. Composite scaffolds incorporating acellular cartilage matrix (ACM) and synthetic polymers have shown particular promise for cartilage tissue engineering applications. However, the present composite scaffold has not been considered as a clinically available application due to insufficient mechanical property or inflammatory response.

**Methods:**

This study presents the composite scaffold composed of ACM and polycaprolactone (PCL) prepared by the low-temperature deposition manufacturing (LDM).

**Results:**

The PCL/ACM scaffold exhibited a Young’s modulus of 462 ± 119 kPa and a compressive yield stress of 592 ± 87 kPa. After 2 weeks in vitro, cell viability presents 92.36% ± 13.41% in PCL/ACM scaffold. Quantification through type II collagen immunofluorescence intensity measurements, exhibited a 1.85-fold increase (p < 0.001) in the PCL/ACM group relative to PCL controls.

**Discussion:**

Through LDM, the ACM was uniformly bond to PCL, resulting in satisfactory mechanical properties of the scaffold. Additionally, the scaffold had a multi-scale structure including microscale pores and nanoscale pores, which increased the porosity of the scaffold. Finally, cartilage-specific extracellular matrix deposition were successfully regenerated *in vivo*.

## Introduction

Cartilage transplantation represents a critical modality in reconstructive surgery, with established clinical applications spanning nasal reconstruction, saddle nose deformity correction, secondary cleft lip nasal deformities, and auricular reconstruction. At present, autologous costal cartilage is the preferred choice for nasal and auricle reconstruction (Fedok, 2016; Song et al., 2023). Despite its clinical predominance, autologous costal cartilage transplantation presents several significant limitations including donor-site morbidity, visible scarring at the donor site, persistent postoperative pain, progressive calcification of remaining rib cartilage (Dong et al., 2022; Meng et al., 2022; Ohara et al., 1997). The development of tissue engineered cartilage provides a new strategy for tissue reconstruction. Previously, tissue-engineered cartilage based on the scaffold made by polyglycolic acid (PGA), polylactic acid (PLA), and polycaprolactone (PCL) has been used in auricle and nasal reconstruction (Xu et al., 2015; Zhou et al., 2018). Nevertheless, the inconsistent levels of postoperative inflammation and deformation have restricted its broader clinical adoption. The underlying causes of this phenomenon may be attributed to the following factors: (1) the residual synthetic polymer materials induced the aseptic inflammatory responses. (2) The absence of a stable extracellular microenvironment compromises chondrocyte proliferation and synthetic activity. (3) The scaffold’s insufficient pore uniformity and microporosity hinder homogeneous chondrocyte distribution and nutrient diffusion. Natural materials exhibit excellent biocompatibility, low toxicity, and can be completely degraded *in vivo* with degradation products without inducing severe inflammatory responses. Nevertheless, scaffolds constructed solely from natural biomaterials frequently exhibit insufficient mechanical strength, thereby compromising their capacity to provide adequate structural integrity and dimensional stability. Composite scaffolds fabricated by combining polymeric materials with natural components can integrate both superior mechanical properties and excellent tissue compatibility, overcoming the limitations of single-component scaffold, becoming the hot spot of tissue engineering (Kundu et al., 2015; Li et al., 2018; Liu et al., 2019).

The acellular cartilage matrix (ACM) is a naturally derived biomaterial capable of closely mimicking the native cartilage extracellular microenvironment. Owing to its excellent histocompatibility, ACM provides an optimal substrate for seed cell adhesion and proliferation. Furthermore, ACM preserves essential structural components of native cartilage tissue, including type II collagen and glycosaminoglycans, while retaining bioactive factors such as transforming growth factor-beta (TGF-β) and insulin-like growth factor (IGF). These inherent biological properties collectively facilitate chondrogenic differentiation and enhance extracellular matrix secretion by chondrocytes (Jiménez-Gastélum et al., 2019; Sutherland et al., 2015; Wang et al., 2015). The composite scaffold composed of artificial polymer synthetic material and ACM is expected to become the ideal tissue engineering cartilage. However, the fabrication of the composite scaffolds imposes more stringent requirements, necessitating simultaneous consideration of the physicochemical properties of both polymeric and ACM to achieve three-dimensional architectures with homogeneous internal porosity. Conventional manual blending methods present inherent challenges in achieving uniformly distributed internal porosity within the scaffold matrix (Wiggenhauser et al., 2019). The advent of three-dimensional (3D) technology has enabled rapid and precise fabrication of scaffolds with precisely controllable pore dimensions (Daly et al., 2021). Jia et al. (2022) reported a layer-by-layer printing approach for fabricating scaffolds using ACM-incorporated hydrogels and PCL. However, this layer-by-layer deposition method fails to achieve microscale material homogeneity, and the thermal processing required for PCL extrusion inevitably compromises the bioactivity of ACM components.

Low-temperature deposition manufacturing (LDM) represents one of the most promising rapid prototyping technologies. The manufacturing process involves non-heating liquefying of materials. Therefore, it can effectively preserve the bioactive components. Furthermore, in addition to controllable macropores, scaffold fabricated by LDM has inter-connected micropores in the deposited lines, which has been widely applied in bone and cartilage tissue engineering (Huang et al., 2018; Chen et al., 2013).

This study aims to fabricate a novel composite scaffold for cartilage tissue engineering by combining PCL and ACM through LDM technique. The LDM technique can prepare the scaffolds without destroying the activity of ACM, and achieve controlled porosity and multi-scale pore structure (Liu et al., 2017; Zhang et al., 2017). This approach shows promising potential as an optimal fabrication strategy for developing composite scaffolds combining polymeric materials with ACM.

## Methods

### Materials and animals

All chemical reagents were supplied by Servicebio (Wuhan, China). Nude mice (female, 5 weeks old) were purchased from Beijing Vital River Laboratory Animal Technology Co., Ltd. (Beijing, China). Primary chondrocytes were isolated from nasal septal cartilage tissue obtained from patients undergoing rhinoplasty that involved partial septal cartilage resection. Acellular cartilage matrix was derived from surplus costal cartilage harvested during autologous costal cartilage-based rhinoplasty procedures. Patients were informed of all procedures and signed consent. All procedures performed in this study were following the ethical standards of the committee of the authors’ institution (certificate number, 2023168). Animal experiments were approved by the Animal Care and Experiment Committee of Plastic Surgery Hospital Institute, Chinese Academy of Medical Science & Peking Union Medical College (certificate number, 202391, Beijing, China).

### Preparation of acellular cartilage matrix

Acellular cartilage matrix was derived from surplus costal cartilage harvested during autologous costal cartilage-based rhinoplasty procedures. The harvested cartilage tissues were mechanically processed through the following steps: (1) Cartilage samples were initially minced into fine particles (<1 mm^3^) using sterile surgical blades. (2) The fragmented tissue was then cryoground into a homogeneous powder using an automated sample grinding system (Shanghai, Jingxin Co., Ltd., China). The cartilage powders were sequentially digested by 0.25% trypsin, nuclease solution (50 U/mL deoxyribonuclease and 1 U/mL ribonuclease A in 10 mM Tris-HCl) and rinsed by 1% Triton X/PBS solution. The DNA content of ACM was measured using the Quant-iT PicoGreen dsDNA Assay Kit (Thermo Fisher Scientific). DNA concentration measurements were performed using a spectrophotometer after digestion and centrifugation of 10 mg ACM with 0.5 mg/mL proteinase K in TE buffer. After the freeze-drying process, the prepared ACM was stored at −20°C condition. The prepared ACM was observed through a scanning electron microscope. Five locations were randomly selected from the obtained ACM samples, and images were acquired using a scanning electron microscope at ×500 magnification. The images were then imported into ImageJ software for dimensional measurement of the ACM.

### Preparation of the scaffold

**FIGURE 1:**
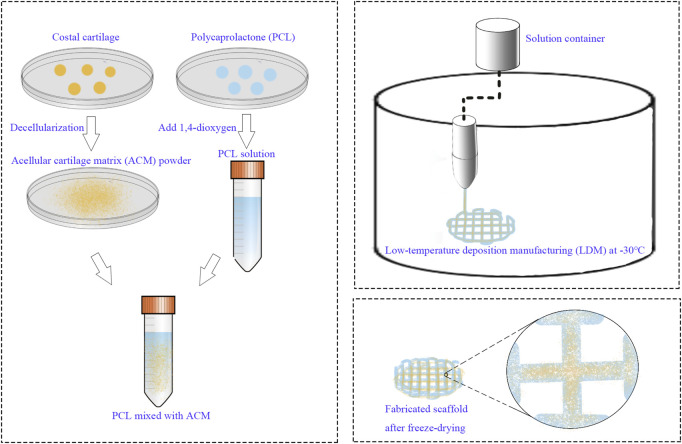
Schematic illustration of the fabrication procedure of the scaffold by LDM system.

A 12.5% (w/v) polycaprolactone (PCL) solution was prepared by dissolving PCL in 1,4-dioxane. ACM powder was subsequently incorporated into the solution at a ratio of 1:5 (ACM:PCL), followed by continuous stirring at 800 rpm and 40°C for 72 h. A pure 12.5% PCL solution, without ACM, served as the control material. The low-temperature deposition manufacturing (LDM) system was configured with a forming chamber temperature of −30°C and a nozzle temperature of 35°C. The scaffold design consisted of a cylindrical structure with a diameter of 10 mm and a height of 3 mm. Following printing, the scaffolds were subjected to lyophilization in a freeze-dryer for 72 h (Figure 1).

### Morphologies and mechanical properties of the scaffold

The morphologies of the scaffolds were characterized via SEM analysis (ZEISS GeminiSEM 300, Germany) at an accelerating voltage of 5 kV (*n* = 5 per group). Before the SEM observation, the samples were sputter-coated with gold for 60 s. Five locations were randomly selected from both group of the scaffold, and images were acquired using a scanning electron microscope at 30× and 5,000× magnification respectively. The images were then imported into ImageJ software for dimensional measurement of the pore and micropores. The compressive properties of the both group scaffolds (*n* = 8 per group) were tested by using an EnduraTEC ELF 3200 (Bose) at dry condition. The diameter and thickness of each scaffold were measured through a vernier caliper. A force transducer measuring 100 N was chosen in an unconstrained uniaxial compression test using a loading rate of 5 mm/s until breaking, the compressive yield strength and the stress strain ratio was recorded and young’s modulus was calculated.

### Cultivation and planting of the seed cells

Nasal septal cartilage specimens were harvested from patients undergoing rhinoplasty with concomitant septal cartilage excision. Under aseptic conditions, the cartilage tissue was sectioned into 1 mm^3^ fragments, followed by enzymatic digestion using 0.2% collagenase type II at 37°C for 8 h. Subsequently, the isolated chondrocytes were cultured in high-glucose Dulbecco’s Modified Eagle Medium (DMEM) supplemented with 10% fetal bovine serum and 1% penicillin-streptomycin-neomycin antibiotic mixture. P2 chondrocytes were utilized as seed cells and suspended in DMEM at a density of 5 × 10^5^ cells/mL. Each scaffold was inoculated with 1 mL cell suspension. Following a 2-week culture period in the medium, cell viability, morphology, and type II collagen expression were assessed via immunofluorescence staining. Cell viability was determined using a live/dead assay, while cytoskeletal morphology was evaluated through phalloidin staining. For type II collagen immunofluorescence analysis, samples were incubated with a rabbit polyclonal anti-human type II collagen primary antibody (1:100 dilution, Abcam), followed by a goat anti-rabbit IgG 594 secondary antibody (1:500 dilution, Abcam). Nuclei were counterstained with DAPI staining solution. For each specimen, four random areas were selected and images were harvested, then images were imported into ImageJ software. Cells counting were conducted to calculate the cell survival rate and the integrated optical density (IOD) values were calculated to quantify the type II collagen expression.

### Histological and immunohistochemical analyses

The scaffolds were embedded subcutaneously in nude mice (*n* = 12) after the scaffolds were seeds with chondrocytes and cultured in the medium for 2 weeks. The PCL/ACM scaffolds were embedded on the left side and the PCL scaffolds were embedded on the other side. Specimens were collected after 4, 8, and 16 weeks respectively (*n* = 4 for each scaffold at each time point). Samples were fixed in 4% paraformaldehyde, embedded in paraffin, sectioned, and stained with hematoxylin-eosin, safranin solid green, and Alcian blue to evaluate the structure and the extracellular matrix deposition. Immunohistochemical analyses were performed to detect the expression of type II collagen. For each specimen, four random areas were selected from the Alcian blue-stained sections and type II collagen immunohistochemical sections, and high-magnification microscopic images were acquired. The images were then imported into ImageJ software. The IOD values were subsequently calculated to quantitatively assess the relative expression levels of glycosaminoglycans (GAGs) and type II collagen.

### Statistical analysis

All statistical analyses were performed with SPSS statistical software (version 26.0; SPSS, Chicago, IL). All graphical representations were generated using GraphPad Prism software (version 9.0). Categorical variables were summarized as number (%) and proportion, while continuous variables were summarized as mean (SD). Values were determined to have a normal distribution using the Shapiro–Wilk test. Differences in the diameter, thickness, pore size, Young’s modulus and the integrated optical density values of immunohistochemical sections of the PCL and PCL/ACM scaffold was invested by ANOVA. The differences in integrated optical density values of immunohistochemical sections on different occasions were analyzed with one-way repeated measures ANOVA test. Results were considered significant when *P* ≤ 0.05.

## Results

### Characterization of acellular cartilage matrix

**FIGURE 2:**
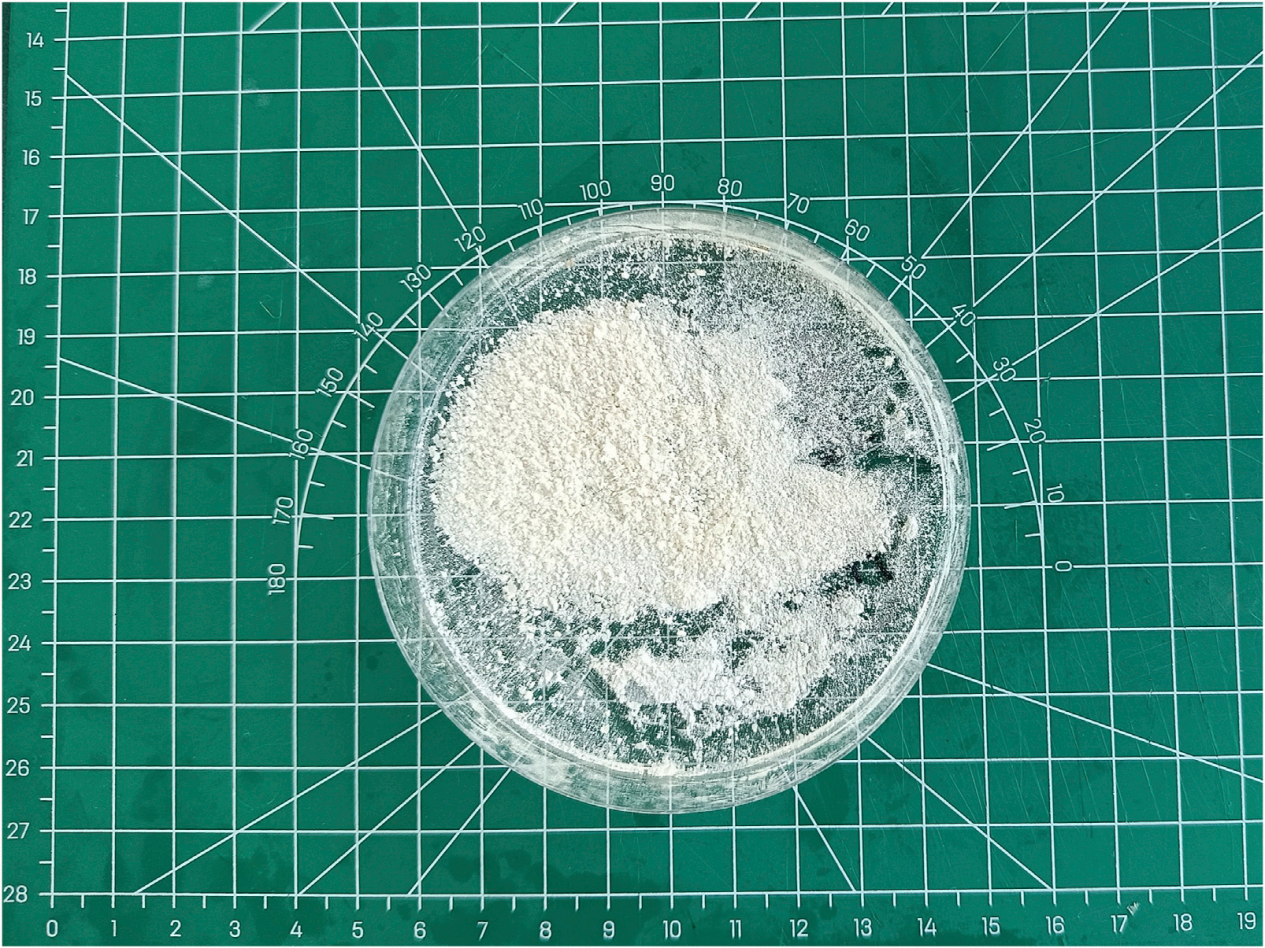
The obtained ACM through procedures of grinding, enzymolysis, and freeze drying.

**FIGURE 3:**
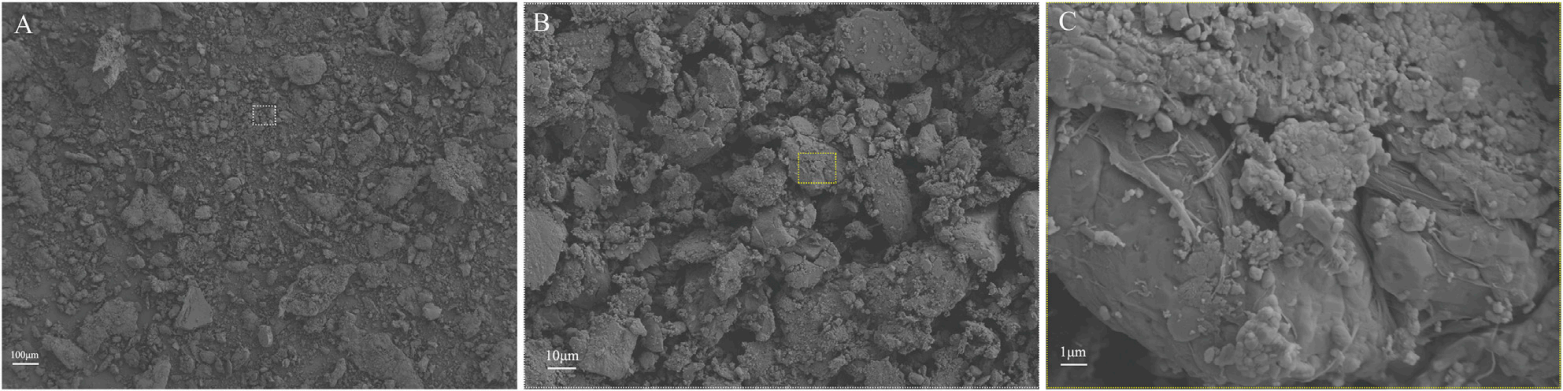
Representative SEM images of ACM acquired at varying magnification levels. **(A)** at 100x magnification; **(B)** at 500x magnification; **(C)** at 5000x magnification.

**FIGURE 4:**
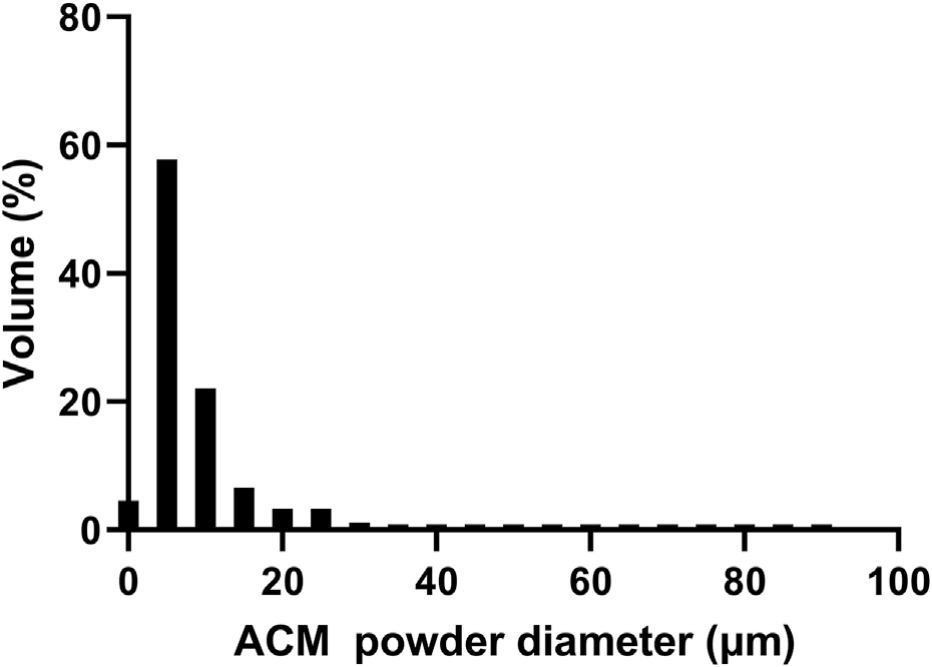
Particle size distribution of the ACM.

Through procedures of grinding, enzymolysis, and freeze drying, the DNA content of the prepared ACM in this study was 15.33 ± 4.04 ng/mg. The obtained ACM exhibited a white powdery morphology (Figure 2). No cellular structures were found under scanning electron microscopy observation, which indicated the decellularization process was relatively thorough. Fiber-like matrix structures were observed at 5,000–10,000 magnification (Figure 3). The diameters of the prepared ACM powders were 8.75 ± 8.45 μm. Figure 4 presents the overall particle size distribution of the obtained ACM powder. However, particles with diameters below 5 μm were not amenable to precise quantification through scanning electron microscopy image due to resolution limitations. Consequently, the actual diameter distribution of ACM particles refers to a progressive increase in particle dimensions.

### Characterization of the scaffold

**FIGURE 5:**
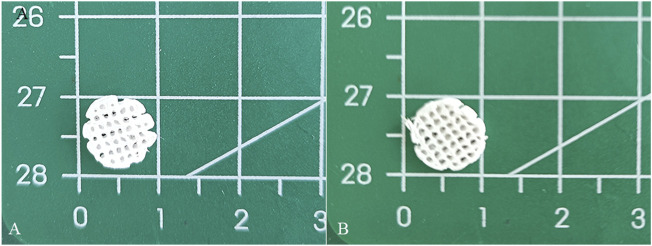
Macroscopic appearance of PCL and PCL/ACM scaffolds fabricated by LDM followed by freeze-drying. **(A)** PCL scaffold; **(B)** PCL/ACM scaffold.

**FIGURE 6:**
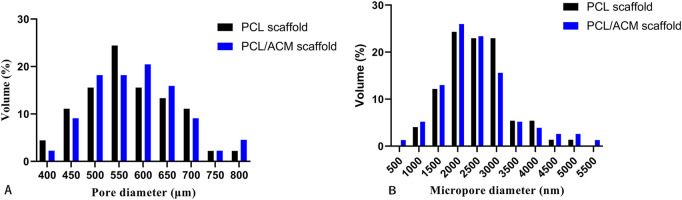
Pore size distribution of PCL and PCL/ACM scaffolds. **(A)** Size and distribution of the macropores; **(B)** Size and distribution of the micropores.

**FIGURE 7:**
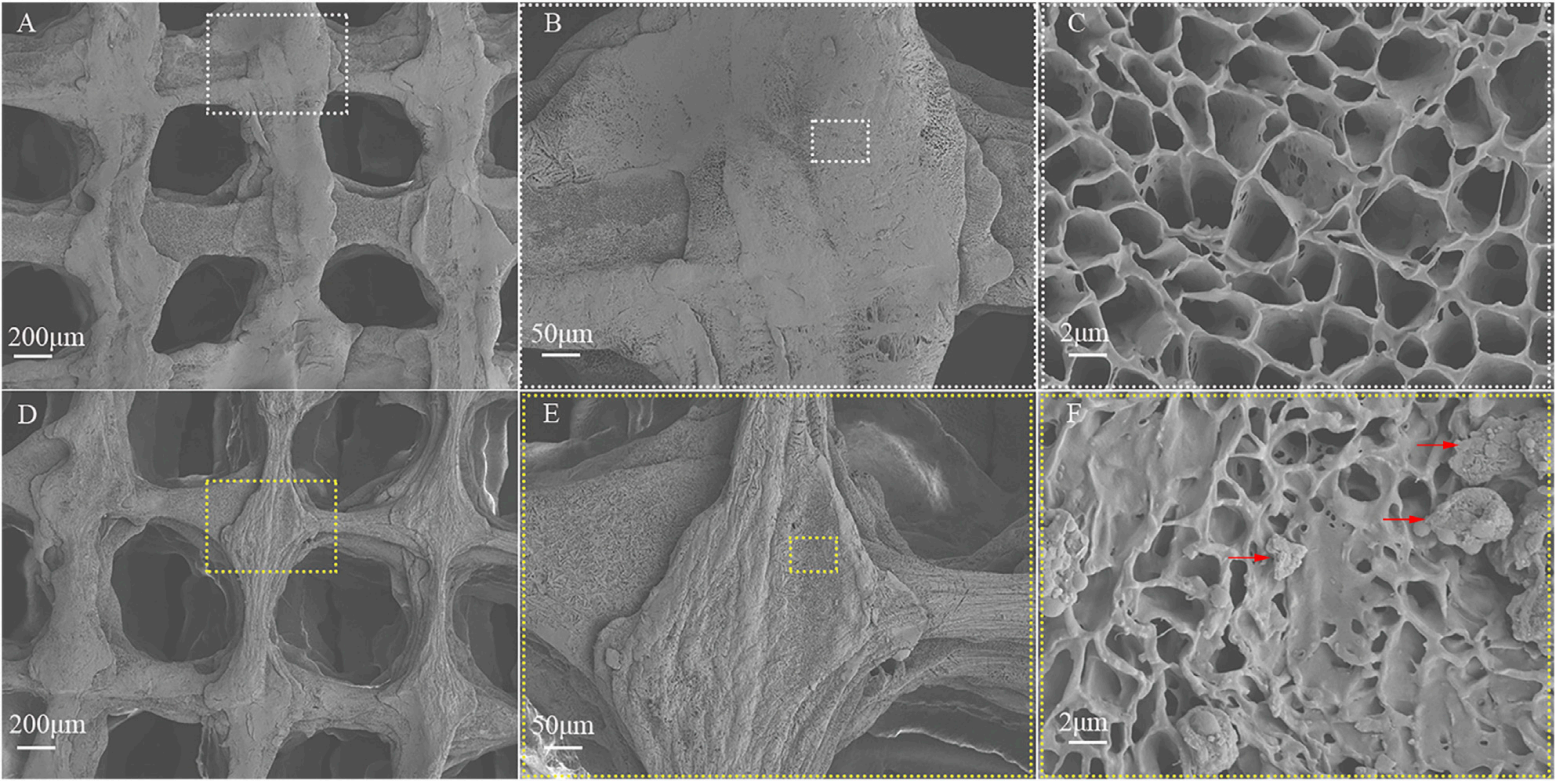
SEM image of the PCL and PCL/ACM scaffolds. **(A)** The PCL scaffold exhibited a muti-scale porous structure at ×30 magnification; **(B)** The extruded strands of PCL exhibited uniform width at ×100 magnification; **(C)** The structure of micropores within the PCL scaffolds was presented at 5,000× magnification; **(D)** The muti-scale porous structure of the PCL/ACM scaffold at ×30 magnification; **(E)** The extruded strands width of PCL/ACM exhibited relative viability at ×100 magnification; **(F)** At 5,000× magnification, ACM particles adherent to the internal scaffold were observed, as indicated by the red arrows.

**FIGURE 8:**
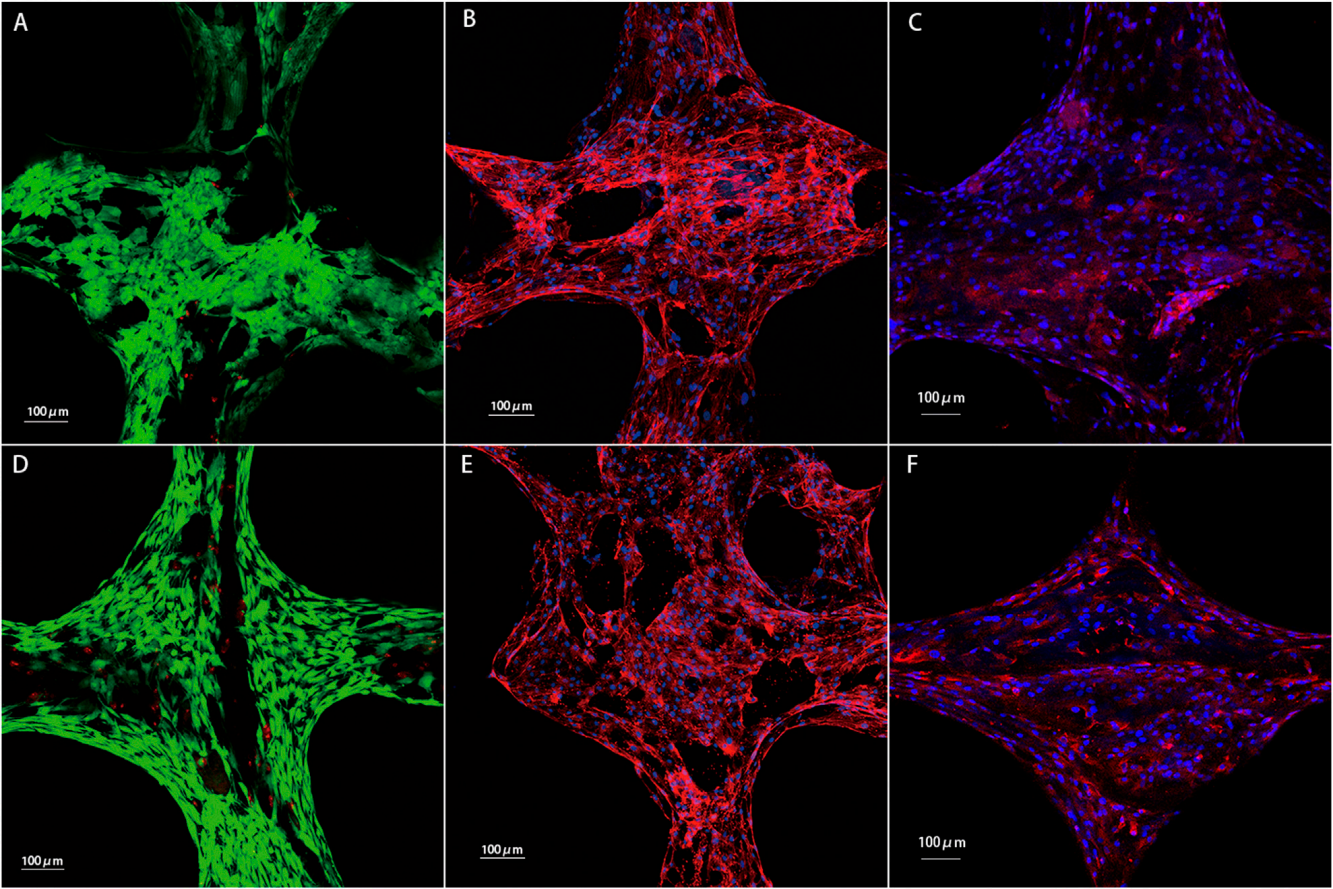
Results of chondrocytes cultured on the PCL and PCL/ACM scaffolds for 2 weeks *in vitro*. **(A)** Cell viability test for chondrocytes on the PCL scaffold. Chondrocytes with good viability were stained green, while dead cells were stained red; **(B)** phalloidin staining for the PCL scaffold; **(C)** Immunofluorescence staining of type II collagen for the PCL scaffold. Type II collagen was stained red and nuclei were stained blue; **(D)** cell viability test for chondrocytes on the PCL/ACM scaffold; **(E)** phalloidin staining for the PCL/ACM scaffold; **(F)** Immunofluorescence staining of type II collagen for the PCL/ACM scaffold.

The diameter and thickness of the prepared PCL scaffold were 9.11 ± 0.26 mm and 2.40 ± 0.40 mm respectively. The diameter and thickness of the prepared PCL/ACM scaffold were 9.19 ± 0.29 mm and 2.29 ± 0.22 mm respectively. There was no significant difference in diameter and thickness between the two groups (Figure 5). Under scanning electron microscopy observation, the porosity of the two groups of the scaffold was relatively uniform, the diameter of pores of PCL scaffold was 573.6 ± 92.3 μm, while that of the PCL/ACM scaffold was 585.4 ± 93.0 μm, with no statistically significant difference between the two groups (*n* = 5 per group) (p = 0.551) (Figure 6A). Under high-magnification of the electron microscopy, microporous structures generated by gas-solid phase separation could be clearly observed, and the attached ACM particles could be observed on the PCL/ACM scaffolds (Figure 7). The diameter of micropores of the PCL scaffold was 2506.1 ± 811.1 nm, and that of the PCL/ACM scaffold was 2456.4.1 ± 984.9 nm, with no statistically significant difference between the two groups (*n* = 5 per group) (p = 0.736) (Figure 6B). According to mechanical testing, the Young’s modulus of the PCL scaffold was 502 ± 127 kPa, with a compressive yield stress of 546 ± 112.61 kPa, while the PCL/ACM scaffold exhibited a Young’s modulus of 462 ± 119 kPa and a compressive yield stress of 592 ± 87 kPa. No statistically significant differences were observed in either Young’s modulus or compressive yield stress between the two groups of scaffolds (Figure 8).

### Cell viability, morphology, and type II collagen immunofluorescence

**FIGURE 9:**
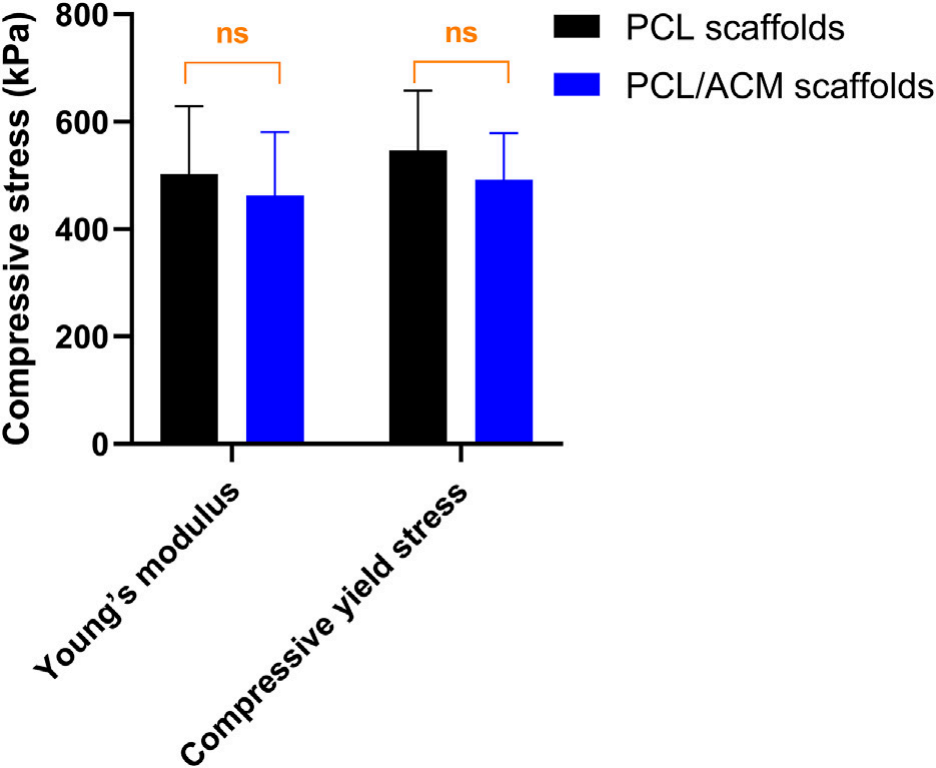
Mechanical testing of PCL scaffold (*n* = 8) and PCL/ACM scaffold (*n* = 8), ns, not significant.

**FIGURE 10:**
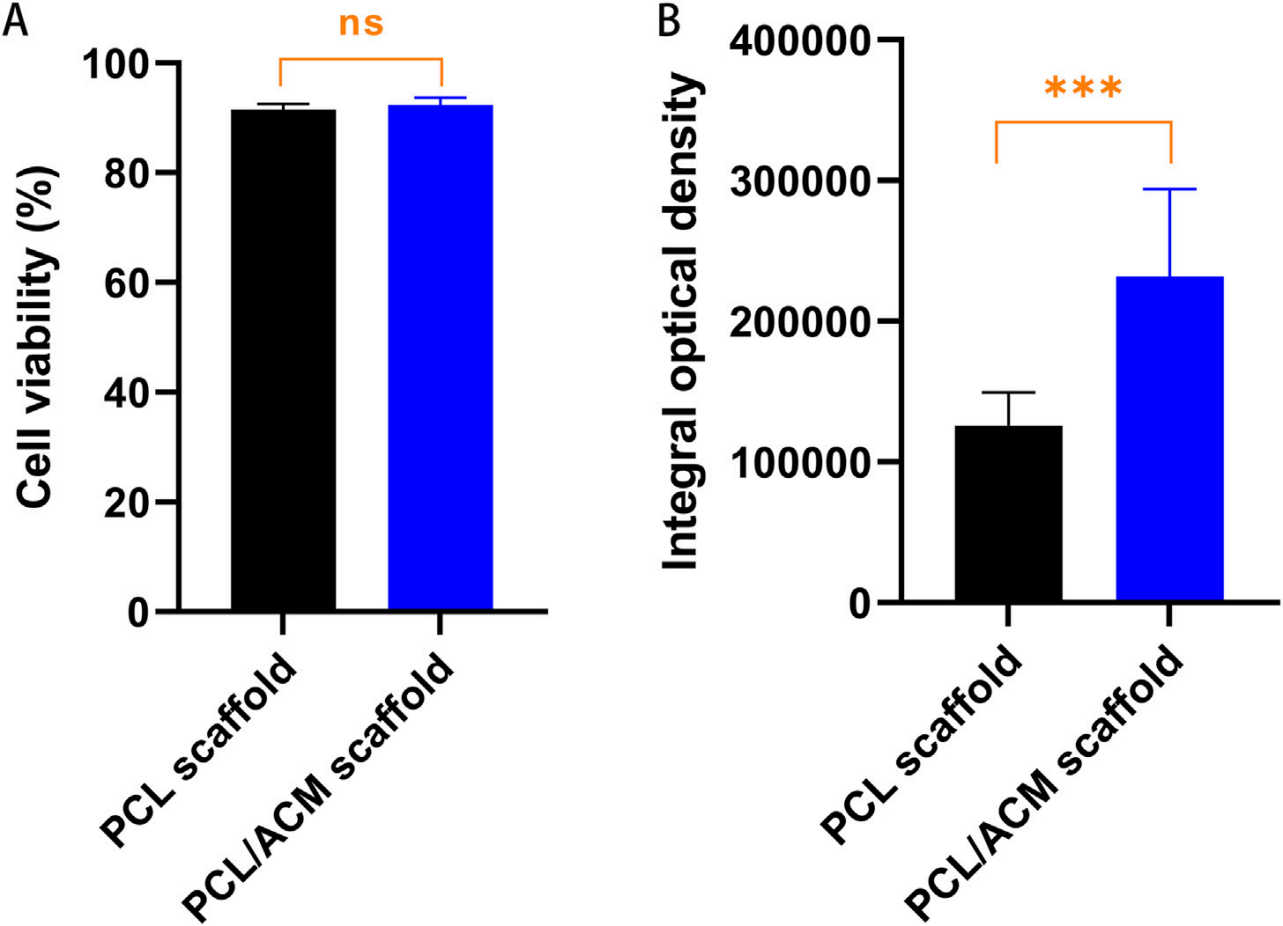
Cell viability and quantification assessment of type II collagen of PCL and PCL/ACM scaffolds 2 weeks of *in vitro* cultivation. **(A)** Cell survival rate of PCL scaffolds (*n* = 3) and PCL/ACM scaffolds (*n* = 3); **(B)** measurements of integral optical density for immunofluorescence of PCL scaffolds (*n* = 3) and PCL/ACM scaffolds (*n* = 3); ns, not significant, ***p < 0.001.

Cell live/death staining, phalloidine staining, and type II collagen immunofluorescence staining were performed after seeding chondrocytes and cultivating for 2 weeks *in vitro* (Figure 9). Cell viability assessment demonstrated excellent survival rates on both scaffold types, which was 91.54% ± 10.10% in PCL scaffold group and 92.36% ± 13.41% in PCL/ACM scaffold. There was no statistical difference between the two groups (p = 0.106) (Figure 10A). Chondrocytes distributed denser and more evenly on the PCL/ACM scaffold than on the PCL scaffold. As visualized by phalloidine staining, comparable cellular morphology was showed between two groups, with typical chondrocyte polygonal morphology maintained in both conditions. Through type II collagen immunofluorescence staining, the distribution of type II collagen on PCL/ACM scaffolds was significantly denser than that on PCL scaffolds. Quantification through type II collagen immunofluorescence intensity measurements, exhibited a 1.85-fold increase (p < 0.001) in the PCL/ACM group relative to PCL controls (Figure 10B).

### *In vivo* evaluation

**FIGURE 11:**
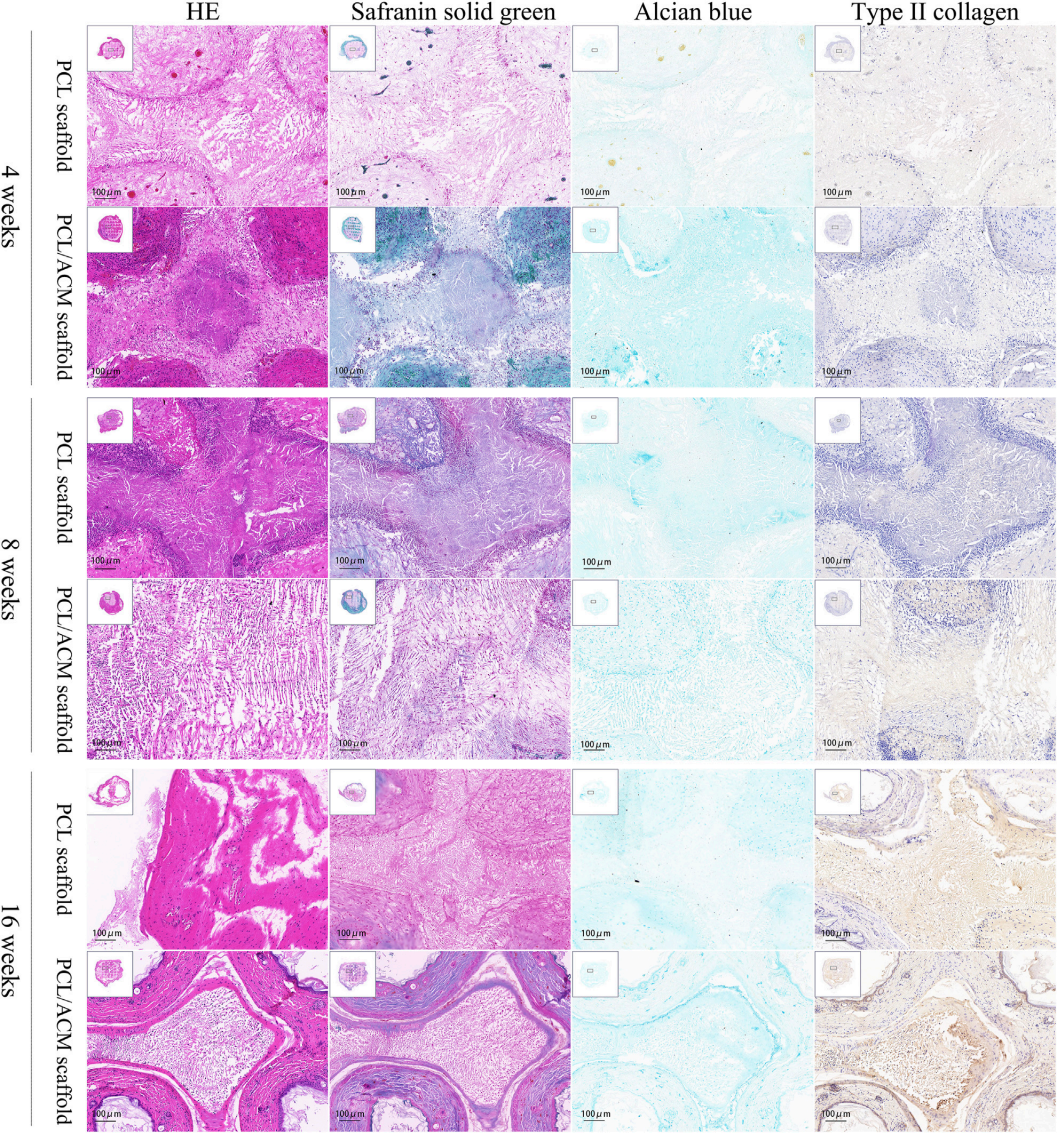
Hematoxylin-eosin, safranin solid green, Alcian blue, type II collagen immunohistochemical staining of PCL scaffold and PCL/ACM scaffold at 4, 8, and 16 weeks cultivation *in vivo*.

**FIGURE 12:**
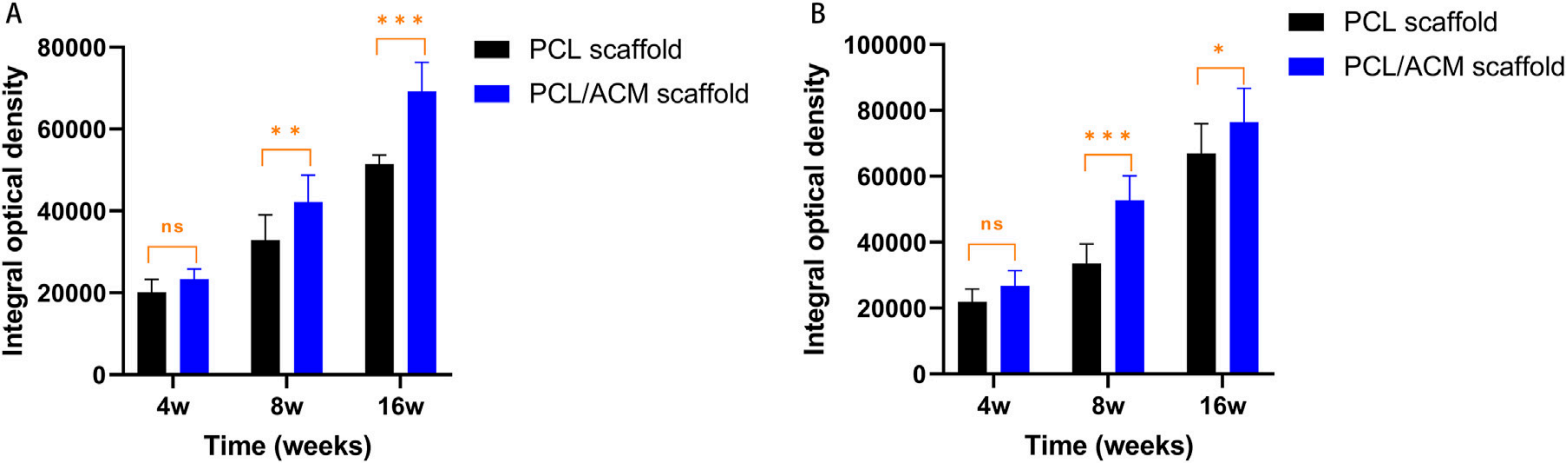
Quantification expression assessment of GAG and type II collagen for PCL scaffold and PCL/ACM scaffold at 4, 8, and 16 weeks cultivation *in vivo*. **(A)** Expression of GAG of both scaffold group (*n* = 4 per group) at 4, 8, and 16 weeks; **(B)** Expression of type II collagen of both scaffold group (*n* = 4 per group) at 4, 8, and 16 weeks; ns, not significant; *p < 0.05, **p < 0.01, ***p < 0.001.

After 2 weeks of *in vitro* seeding and cultivation of chondrocytes on the scaffolds, the scaffolds from both groups were subcutaneously implanted into nude mice. Specimens were harvested at 4, 8, and 16 weeks post-implantation (Figure 11). At the 4-week time point, no significant scaffold degradation was observed in either group. Tissue formation was evident within the scaffold pores. Through assessment of the integral optical density, there was no statistical significant difference in the expression of glycosaminoglycan (GAG) (p = 0.330) and type II collagen (p = 0.296) compared to PCL scaffolds. By 8 weeks, quantification assessment of GAG and type II collagen content presented 1.63-fold increase (p = 0.001) and 1.53-fold increase (p = 0.018) respectively in PCL scaffold group compared to that of 4 weeks point. While the GAG and type II collagen content presented 1.81-fold increase (p < 0.001) and 1.84-fold increase (p < 0.001) respectively in PCL scaffold group compared to that of 4 weeks point. There was significant difference in the expression of GAG (p = 0.007) and type II collagen (p < 0.001) between the two scaffold groups. At 16 weeks, the majority of the scaffold material had degraded. Abundant chondrocyte lacunae were observed in both groups, with a more pronounced presence in the PCL/ACM group. Safranin O-fast green staining revealed distinct red-stained cartilage matrix in both groups, particularly surrounding the lacunae. For PCL scaffold, quantification assessment of GAG and type II collagen content exhibited 1.57-fold increase (p < 0.001) and 1.99-fold increase (p < 0.001) respectively compared to that at 8 weeks. For PCL/ACM scaffold, quantification assessment of GAG and type II collagen content exhibited 1.64-fold increase (p < 0.001) and 1.45-fold increase (p < 0.001) respectively compared to that at 8 weeks. The PCL/ACM scaffold group demonstrated significantly higher levels of GAG (p < 0.001) and type II collagen (p = 0.047) components compared to PCL scaffold group (Figure 12).

## Discussion

Because of the similar components and composition to native tissues, ACM has been expected as the most promising biomaterial in tissue-engineered cartilage. DNA content was usually used to evaluate the residual cellular components to determine whether the decellularization process is complete. Previous studies suggested the DNA content should be lower than 50 ng/mg (Gomes et al., 2015). In our study, the DNA content of the prepared ACM was 15.33 ± 4.04 ng/mg which was significantly lower than the suggested standard. In addition, the scanning electron microscopy observation results also confirmed the decellularization process.

After grinding, the diameters of the prepared ACM powders were less than 25 μm. Based on our experimental results, the ratio of ACM to PCL was optimized at 1:5 in this study. It is noteworthy that systematic investigation of varying ACM/PCL ratios was precluded due to recurrent nozzle clogging during the printing process at elevated ACM concentrations, which significantly compromised scaffold fabrication fidelity. This operational limitation consequently led to unsatisfactory mechanical performance of the resultant scaffolds. Further refinement of the ACM grinding process to obtain more homogeneous and finer ACM powder might potentially allow for higher ACM proportions without compromising scaffold formation. In addition, previous literature has reported the methods to increase the hydrophilia of ACM through enzymolysis and modification, which also provided new ideas for the combination of ACM with polymer materials (Chen et al., 2020; Ju et al., 2023; Wu et al., 2021).

Scaffolds made solely by ACM had the problem of insufficient mechanical performance. Composite scaffold combined with artificial polymer material could effectively improve the mechanical properties of the scaffold (Yang et al., 2017). Utilizing the thermoplastic characteristics of materials, three-dimensional architectures with high precision can be efficiently fabricated through 3D printing techniques, thereby creating a biomimetic microenvironment conducive to cellular attachment and proliferation. However, most synthetic polymeric materials exhibit relatively high melting points. Even PCL which possesses a comparatively lower melting temperature, typically requires processing temperatures exceeding 65°C during melt fabrication. At this thermal threshold, critical biological components such as collagen proteins undergo denaturation, while the three-dimensional architecture of most natural materials experiences structural collapse and irreversible conformational changes (Gruber et al., 2019). The low-temperature deposition 3D printing process fabrication process was conducted at −30°C. Excessively low temperatures significantly increase the viscosity of the PCL solution, thereby elevating the risk of nozzle clogging during deposition. Conversely, elevated temperatures prolong the solidification time or may even prevent proper material formation, ultimately compromising the structural stability of the scaffolds. Through this fabrication process, ACM and PCL could be uniformly combined without damaging the biological activity of ACM. The macropore size could be controlled by setting the parameters of the LDM program. A pervious study has confirmed the feasibility of 500 μm pore size of macropore for the attachment and proliferation of chondrocytes (Liu et al., 2017). In addition, through the freeze-drying process, micropores formed by the separation of the gas-solid phase, which was beneficial for nutrient exchange in the scaffold (Crapo et al., 2011; Sun et al., 2023).

The decellularization process employed physical, chemical, and enzymatic methods to disrupt cellular structures, remove DNA and proteins, and eliminate antigenic components from the tissue (Bastiaansen-Jenniskens et al., 2008). Following decellularization, components such as type II collagen and glycosaminoglycan were relatively preserved. Collagen was reported to account for the main dry weight of human cartilage, and insufficient collagen content has been has been associated with the inferior mechanical and functional properties characteristic of tissue-engineered cartilage constructs (Whitney et al., 2018; Chang et al., 2014). Previous studies have demonstrated that ACM powders had the potential of promoting type II collagen expression (Yu et al., 2022). In this study, after cell seeding and 2 weeks of *in vitro* culture, the PCL/ACM scaffolds exhibited significantly higher type II collagen expression compared to PCL-only scaffolds. Notably, collagen deposition was particularly pronounced in the pericellular regions surrounding chondrocytes. This observation may be attributed to two factors: (1) the inherent presence of type II collagen within the ACM component of the PCL/ACM scaffold, and (2) the potential stimulatory effect of ACM on chondrocyte-mediated type II collagen synthesis. These findings were further corroborated by immunohistochemical analysis of type II collagen in specimens obtained from long-term *in vivo* culture.

This study presented the composite scaffold for tissue-engineered cartilage through low-temperature deposition 3D printing. However, the current study has some limitations and needs further improvement. First, different ACM to PCL ratio was not available to be investigated to explore the trends in mechanical properties and tissue compatibility of scaffolds. Second, the study lacked quantitative analysis of scaffold thermal stability to evaluate changes in thermal properties following low-temperature fabrication. Additionally, the mechanical testing of scaffolds in this study was conducted following freeze-drying, which might introduce discrepancies compared to their performance under physiological conditions within human tissues.

## Conclusion

In this study, a composite scaffold consisting of PCL and ACM was fabricated by LDM. The LDM could effectively protect the biological activity of ACM. In addition, the scaffold had a multi-scale structure including microscale pores and nanoscale pores, which increased the porosity of the scaffold. The composite scaffold had satisfactory mechanical properties. Most importantly, ACM powders were successfully mixed with PCL and uniformly distributed as scaffold material. The feasibility and superiority of the scaffold were confirmed through *in vitro* and *in vivo* culture, which was expected to be widely used in tissue-engineered cartilage in the future.

## Data Availability

The original contributions presented in the study are included in the article/supplementary material, further inquiries can be directed to the corresponding authors.
